# Age, left atrial dimension and arterial stiffness after external cardioversion of atrial fibrillation. A vascular component in arrhythmia maintenance? Results from a preliminary study

**DOI:** 10.1007/s40520-013-0173-z

**Published:** 2013-11-24

**Authors:** Stefano Fumagalli, Debbie Gabbai, Besmir Nreu, Anna T. Roberts, Serena Boni, Alice Ceccofiglio, Stefania Fracchia, Samuele Baldasseroni, Francesca Tarantini, Niccolò Marchionni

**Affiliations:** Intensive Care Unit and Geriatric Arrhythmology Unit, Division of Geriatric Cardiology and Medicine, and Research Unit of Medicine of Aging, Department of Experimental and Clinical Medicine, University of Florence and AOU Careggi, Viale Pieraccini, 6, 50139 Florence, Italy

**Keywords:** Arterial stiffness, Atrial fibrillation, CAVI, Elderly, External cardioversion, Left atrium diameter

## Abstract

**Background and Aims:**

Atrial fibrillation (AF) is the most frequent arrhythmia in elderly patients. Aims of this study were to evaluate the predictors of arterial stiffness after external cardioversion (ECV) of AF and to establish whether a link exists between vascular properties and left atrial diameter (LAD).

**Methods:**

We studied 33 patients (age 73 ± 12 years). After 5 h from ECV of persistent AF, an echocardiogram was recorded and arterial stiffness was evaluated with cardio-ankle vascular stiffness index (CAVI).

**Results:**

In multivariate analysis (*R* = 0.538, *p* = 0.006), CAVI (mean 9.60 ± 1.63) increased with age (*p* = 0.018) and with an AF length ≤3 months (*p* = 0.022). LAD was significantly related to CAVI (*p* = 0.007) even after adjustment for interventricular septum thickness (*p* = 0.018) (*R* = 0.574, *p* = 0.002).

**Conclusions:**

In patients with AF, immediately after ECV, arterial stiffness is associated with age and AF length, and could represent an important factor for left atrium remodeling and, therefore, for AF maintenance.

## Introduction

Atrial fibrillation (AF) is the most common sustained arrhythmia in the elderly [1]; its prevalence is justified by cardiovascular aging and the age-related increase of comorbidity [1–3]. Arterial stiffness (AS) also increases with age and predicts coronary heart disease, stroke and mortality [4]. The endpoint of this study was to define the clinical determinants of AS in subjects with persistent AF immediately after external cardioversion (ECV). Furthermore, we assessed the existence of a link between left atrium (LA) diameter (LAD) and AS.

## Methods

### Patients and procedures

We enrolled all patients admitted to a day-hospital for ECV of persistent AF between January and June 2013; the only exclusion criterion was non-restoration of SR. The protocol was approved by an institutional committee and conforms to the 1975 Declaration of Helsinki ethical guidelines; patients gave their consent to participate. ECV was performed using a biphasic defibrillator, after a 4-week period of effective oral anticoagulation [1]. An echocardiogram was digitally recorded 5 h after the procedure.

### Measure of AS

The cardio-ankle vascular index (CAVI) [5], a measure of AS, independent from instantaneous systolic and pulse pressure, was determined using VaSera VS-1500N (Fukuda Denshi, Japan) after 20 min of rest in supine position. During the continuous recording of EKG and heart sounds, right and left upper and lower extremity arterial pressure was obtained with the oscillometric method. Pulse wave velocity (PWV) was calculated dividing the distance from the aortic valve to the ankle artery (an indirect measure derived from the height of the patient) by the sum of two time intervals [(1) aortic valve closing sound—notch of the brachial pulse wave; (2) rise of the brachial pulse wave—rise of the ankle pulse wave] [5]. CAVI was then computed using the following equation:

$$ {\text{CAVI}} = a \times \left[ {\left( {2\rho /\varDelta P} \right) \times \ln \left( {P_{\text{s}} /P_{\text{d}} } \right) \times {\text{PWV}}^{2} } \right] + b $$

where *P*_s_/*P*_d_ are systolic/diastolic pressures, Δ*P* is “*P*_s_–*P*_d_”, ρ is blood density, and *a*/*b* are constants [5]. CAVI was measured after SR restoration to avoid bias due to different RR intervals [5].

### Statistical analysis

IBM SPSS for Windows (ver. 20) was used for statistical analysis. Continuous variables were expressed as mean ± SD, categorical variables as numbers with percentages. Student’s *t* test and analysis of variance were used to compare continuous variables. The association between categorical variables was evaluated with *χ* ^2^ test. Linear regression analysis models were used to define univariate and multivariate predictors of CAVI and LAD. A *p* value <0.05 was considered statistically significant.

## Results

### Patients’ description

Thirty-three patients underwent ECV (Table 1). Median AF length was 3 months (range 1–18). Right arm arterial pressure and right ankle-brachial index were 140 ± 16/85 ± 11 mmHg and 1.10 ± 0.15, respectively, with no difference between right- and left-side measurements. Thromboembolic risk (CHADS_2_ score) was moderate in 72.7 % and high in 18.3 % of cases. Beta-blockers, ACE-inhibitors or angiotensin receptor blocking agents were the most frequently used cardiovascular drugs (Table 1). There was no difference between right and left CAVI (9.60 ± 1.63 and 9.56 ± 1.64, respectively; *p* = 0.78). Therefore, only the results related to right CAVI will be presented.

**Table 1:** Patients’ clinical characteristics

(*n* = 33)		Range
Age (years)	73 ± 12	35–90
Men (*n*, %)	28 (84.8)	
Height (cm)	174 ± 9	150–192
Weight (Kg)	82 ± 15	56–138
BMI (Kg/m^2^)	27.0 ± 5.0	19.8–48.9
Present smokers (*n*, %)	7 (21.2)	
CAD (*n*, %)	6 (18.2)	
CHF (*n*, %)	11 (33.3)	
CKD (*n*, %)	5 (15.2)	
CVD (*n*, %)	2 (6.1)	
Diabetes (*n*, %)	4 (12.1)	
Dyslipidemia (*n*, %)	12 (36.4)	
Hypertension (*n*, %)	22 (66.7)	
LAF/brady-tachycardia syndrome (*n*, %)	6 (18.2)	
Valvular disease (*n*, %)		
Aortic regurgitation	7 (21.2)	
Aortic stenosis/prosthesis	4 (12.1)	
Mitral regurgitation	17 (51.5)	
CHADS_2_ (score)	1.8 ± 1.3	0–5
Pulse Pressure (mmHg)	57 ± 13	34–95
LAD (mm)	50 ± 7	35–63
Interventricular septum (mm)	10 ± 1.5	7–12
LVEDD (mm)	56 ± 9	43–86
LVESD (mm)	39 ± 10	24–71
LVEF (%)	56 ± 14	20–79
Hemoglobin (g/dL)	14.0 ± 1.7	11.0–17.3
Creatinine (mg/dL)	0.9 ± 0.3	0.4–1.6
ACE-I/ARB (*n*, %)	27 (81.8)	
β-blockers (*n*, %)	25 (75.8)	
Ca-antagonists (*n*, %)	5 (15.2)	
Digoxin (*n*, %)	15 (45.5)	
Statins (*n*, %)	9 (27.3)	

*BMI* body mass index, *CAD* coronary artery disease, *CHF* chronic heart failure, *CKD* chronic kidney disease, *CVD* cerebro-vascular disease, *Interventricular septum* Interventricular septum thickness, *LAF* lone atrial fibrillation, *Brady-Tachycardia Syndrome* bradycardia-tachycardia syndrome, *Prosthesis* biological or mechanical aortic prosthetic valve, *LAD* left atrium end-systolic diameter, *LVEDD/LVESD* left ventricular end-systolic/end-diastolic diameter, *LVEF* left ventricular ejection fraction, *ARB* angiotensin receptor blocking agents, *Ca-antagonists* dihydropyridine Ca-antagonists

### CAVI determinants

In univariate analysis, CAVI was significantly associated with age (*R* = 0.390, *p* = 0.025), BMI (*R* = 0.364, *p* = 0.037), the presence of mitral regurgitation (yes: 10.3 ± 1.4 vs. no: 8.9 ± 1.6, *p* = 0.012) and an AF length ≤3 months (yes: 10.1 ± 1.5 vs. no: 8.9 ± 1.5, *p* = 0.031). CAVI was not affected by gender or current smoking status, nor did we find any link with arterial hypertension, CHF, chronic renal failure, coronary artery and cerebrovascular disease, diabetes, dyslipidemia or other causes of AF. Heart rate, systolic, diastolic and pulse pressure, hemoglobin concentration and drug therapy did not correlate with CAVI. Interventricular septum thickness, left ventricular (LV) diameters, and LVEF were not associated with CAVI, but LAD was (*R* = 0.435, *p* = 0.011). A multivariate regression analysis model (*R* = 0.538, *p* = 0.006) showed that age (*β* = 0.05 ± 0.02, 95 % CI = 0.01–0.09, *p* = 0.018) and an AF length ≤3 months (*β* = 1.20 ± 0.50, 95 % CI = 0.18–2.22, *p* = 0.018) were significantly related to CAVI, while BMI lost its statistical significance (*p* = 0.11).

### LAD determinants

**Fig. 1:**
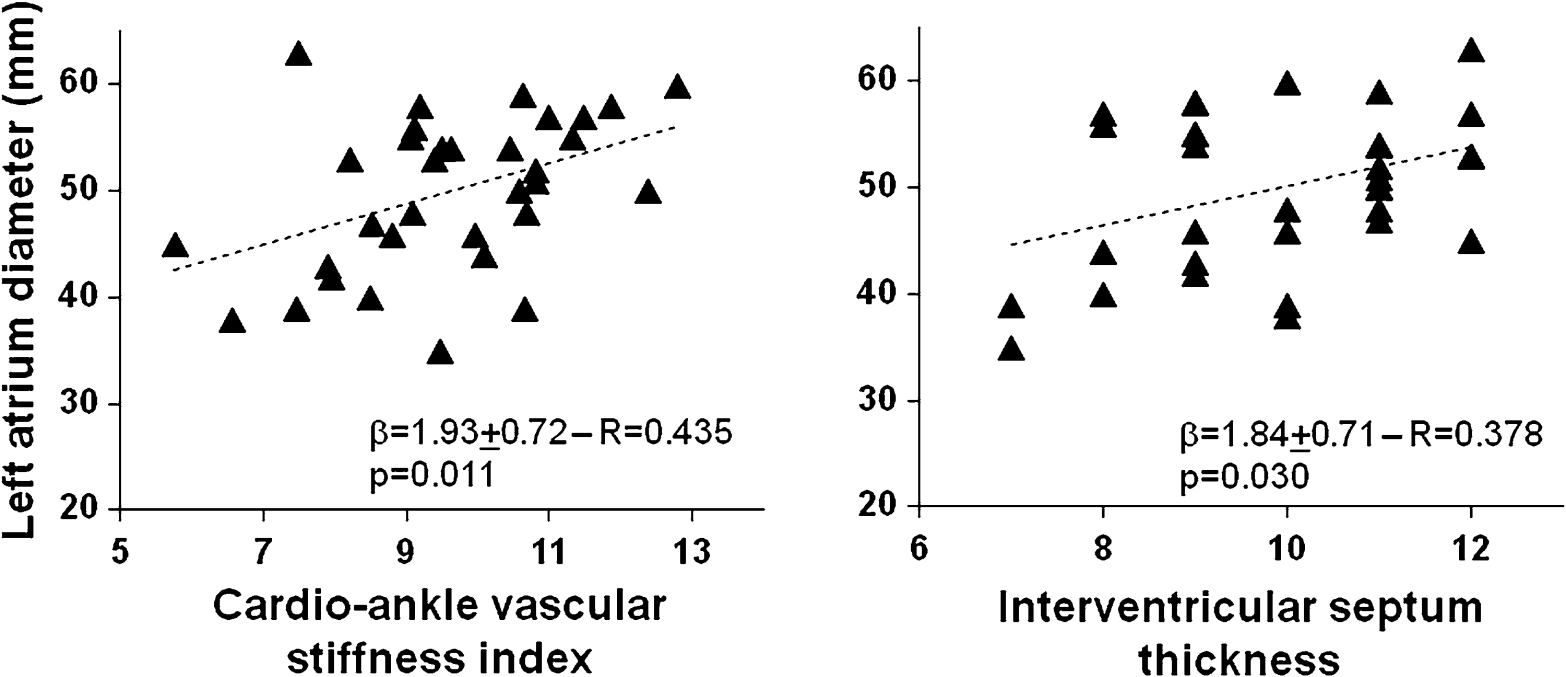
Association between left atrium diameter and Cardio-Ankle Vascular Index (*left*) and interventricular septum thickness (*right*) in patients with persistent atrial fibrillation evaluated after successful external cardioversion (univariate regression analysis model)

LAD was not associated with age, gender, BMI, comorbid conditions, mitral regurgitation, AF length, drugs, arterial pressure, LV diameters, LVEF or hemoglobin concentration. Apart from CAVI, only interventricular septum thickness showed a direct association with atrial dimensions (*R* = 0.378, *p* = 0.030). A multivariate model (overall *R* = 0.574, *p* = 0.002) demonstrated that both interventricular septum thickness (*β* = 1.82 ± 0.73, 95 % CI = 0.34–3.30, *p* = 0.018) and CAVI (*β* = 1.92 ± 0.66, 95 % CI = 0.56–3.27, *p* = 0.007) were predictors of LAD (Fig. 1).

## Discussion

The results of this preliminary study confirm the existence of a direct relationship between CAVI and age, also in patients treated with ECV for persistent AF. Interestingly, a shorter length of AF seems to be associated with higher CAVI values. We also showed that AS, together with interventricular septum thickness, is a significant determinant of LAD.

The inverse correlation between CAVI and duration of AF, observed in the first few months after arrhythmia development, might be attributed to the increase in afterload due to the early effects on cardiac performance associated with the loss of atrial systole [6].

Few data exist aimed at clarifying the interaction among AF, LAD and AS, and results are sometimes conflicting. In a previous study, Reiffel [7] found that AS and LV hypertrophy did not predict the risk of AF in hypertensive patients and concluded that the arrhythmia is generated by complex mechanisms induced by atrial hypertension. On the contrary, Lantelme and colleagues [8] showed that in hypertensive patients LAD correlated with PWV or pulse pressure, suggesting that AF could be a link between AS and stroke. Finally, patients with obstructive sleep apnea showed a direct correlation between LAD and AS [9]. LA and LV act in synergy; atrial volume has been proven to express the severity of ventricular diastolic dysfunction in patients without atrial arrhythmias or valvular heart disease [10]. However, the association of LAD with diastolic dysfunction cannot fully explain our results. In fact, CAVI remained independently correlated with atrial dimensions even after adjustment for interventricular septum thickness, a parameter linked with altered LV relaxation [11]. A possible explanation of the link between AS and LAD we found in this study could be represented by mitral regurgitation, present in 51.5 % of our patients and associated with higher CAVI values. In addition, AS seems to promote inflammation, vascular smooth muscle cell contraction, and stretch-induced release of metalloproteinase-12 in atrial tissues [12]. To further stress the importance of non-mechanical factors in the complex network linking LA, LV and arterial properties, a recent review suggests that comorbidities can produce coronary micro-vascular dysfunction, mediated by inflammation, ultimately leading to myocardial stiffness progression [13]. All these data support the existence of a connection between AS and AF.

### Study limitations

The study is preliminary and the sample is small, with only 5 women. The echocardiogram was recorded 5 h after effective ECV; yet, current evidence seems to suggest that such a short period of SR is unable to revert the electrical and structural changes which characterize atrial remodeling [14]. Finally, our results might have been different had we performed pharmacological cardioversion; however, ECV is the treatment of choice for AF lasting >48 h [1].

In conclusion, the results of our preliminary study support the hypothesis that age-related AS is independently associated with atrial size in patients with persistent AF. Therefore, the concept that “atrial fibrillation begets atrial fibrillation” [15] might rely also on a “vascular” component. Further studies are needed to confirm present findings and to clarify if AS may exert a role in arrhythmia recurrence.
